# Lysozyme as an alternative to growth promoting antibiotics in swine production

**DOI:** 10.1186/s40104-015-0034-z

**Published:** 2015-08-13

**Authors:** W. T. Oliver, J. E. Wells

**Affiliations:** USDA, ARS, U.S. Meat Animal Research Center, P. O. Box 166, Clay Center, NE 68933-0166 USA

**Keywords:** Antibiotics, Gastrointestinal, Lysozyme, Microbiota, Review, Swine

## Abstract

Lysozyme is a naturally occurring enzyme found in bodily secretions such as tears, saliva, and milk. It functions as an antimicrobial agent by cleaving the peptidoglycan component of bacterial cell walls, which leads to cell death. Antibiotics are also antimicrobials and have been fed at subtherapeutic levels to swine as growth promoters. These compounds benefit swine producers by minimizing production losses by increasing feed efficiency and decreasing susceptibility to bacterial infection and disease. This manuscript reviews the knowledge of the effects of lysozyme, as compared to traditional subtherapeutic antibiotics in swine feed, on pig performance and health. It is clear from decades of studies that antibiotic use in feeds increases pig performance, particularly in the nursery. Similarly, lysozyme, as a feed additive, increases growth and feed efficiency. While the mechanism by which antibiotics and lysozyme improve performance is not clearly understood, both of these feed additives improve gastrointestinal health, improve the metabolic profile, and alter the gastrointestinal bacteria ecology of swine. Therefore, lysozyme is a suitable alternative to growth-promoting subtherapeutic antibiotic use in swine feed.

## Introduction

Antimicrobials have been fed at subtherapeutic levels to swine as growth promoters for more than 60 years, and the majority of pigs produced in the U.S. receive antimicrobials in their feed at some point in their production cycle. These compounds benefit swine producers by minimizing production losses by increasing feed efficiency and decreasing susceptibility to bacterial infection and disease [1]. Wells et al. [2] observed 62 % prevalence for *Salmonella* in swine prior to the growing phase of production, and this number decreased to less than 15 % after 8 weeks on diets containing chlortetracycline, a broad based antimicrobial. In addition, increased *Campylobacter* shedding is associated with reduced performance in growing pigs [3]. Therefore, a reduction in pathogen shedding due to antibiotic use appears to be associated with increased animal performance. However, in recent years, foreign and domestic markets have been pressuring swine producers to reduce or remove antimicrobials from their diets.

Lysozyme is a 1,4-β-N-acetylmuramidase that enzymatically cleaves a glycosidic linkage in the peptidoglycan component of bacterial cell walls, which results in the loss of cellular membrane integrity and cell death [4]. In addition, hydrolysis products are capable of enhancing immunoglobulin A (IgA) secretion, macrophage activation, and rapid clearance of bacterial pathogens [5, 6]. These data indicate that lysozyme may be a viable alternative to antibiotics in diets fed to swine.

Until recently, the literature pertaining to lysozyme as a feed additive was limited to studies using transgenic vectors to deliver lysozyme. These studies have shown changes in metabolite profiles [7], intestinal microbiota [8], and intestinal morphology [9] in pigs fed milk from transgenic goats expressing human lysozyme in their mammary gland. In addition, Humphrey et al. [10], reported that diets supplemented with transgenic rice expressing lysozyme had antibiotic-like properties when fed to chicks. While these reports are encouraging, the delivery of lysozyme from transgenic goats’ milk or transgenic rice is problematic in a swine production setting. However, recent research with egg-white lysozyme showed a performance benefit when fed to young pigs [11–13].

## Lysozyme sources and current use

Before discovering penicillin, Alexander Fleming discovered the enzyme lysozyme based on the ability of nasal secretions to prohibit bacterial growth [14]. Lysozyme is a naturally occurring enzyme found in bodily secretions such as tears, saliva, and milk. It functions as an antimicrobial by enzymatically cleaving a glycosidic linkage of bacterial cell walls peptidoglycan, which leads to cell death [4]. Lysozyme is found in many biological organisms from bacteria and fungi to animal bodily secretions and tissues [15, 16]. Lysozyme is an important defense mechanism and is considered a part of the innate immune system in most mammals [17], and is also an important component of human breast milk [18]. However, due to its very low concentration in sow milk (<0.065 μg/mL), lysozyme is not believed to play a major role in the prevention of infection in suckling pigs.

*In vitro*, lysozyme is generally considered effective against some Gram-positive bacteria, but ineffective against Gram-negative bacteria [19]. However, lysozyme, perhaps indirectly, can affect Gram-negative bacteria *in vivo* [11, 20]. Due to these antimicrobial properties, lysozyme has been used effectively in the food industry [21]. For example, it has been used in the cheese industry to prevent late blowing [22, 23]. Lysozyme has also been used as a preservative for other fresh foods [19], including controlling meat spoilage [24].

Lysozyme is not currently used extensively as a feed additive in the animal industry. However, its effectiveness on pigs has been evaluated in different models. Until recently, the literature pertaining to lysozyme as a feed additive was limited to studies using milk from transgenic organisms or transgenic rice to produce and deliver the enzyme. Human lysozyme has been expressed in the milk of pigs [25], mice [26], and goats [8] as models for human medicine. Subsequent studies using transgenic goats’ milk suggested that lysozyme could be used as a feed antimicrobial. These studies have shown changes in metabolite profiles [7], intestinal microbiota [8], and intestinal morphology [9] in pigs fed milk from transgenic goats expressing human lysozyme in the mammary gland. Diets supplemented with transgenic rice expressing human lysozyme also improved the performance of chicks [10]. These experiments were not designed to evaluate lysozyme as a feed additive. However, results from recent experiments have shown that lysozyme sourced from chicken eggs (Neova Technologies; Abbotsford, Canada) improved growth rate and intestinal morphology and reduced *Campylobacter* shedding in both 10-day-old pigs consuming a milk diet [11] as well as nursery pigs [12, 13, 20]. In addition, Nyachoti et al. [27] reported the same source of lysozyme alleviated the piglet response to an oral challenge of *Escherichia coli* K88.

## Lysozyme as a feed additive

### Performance

The use of antibiotics in livestock feed is well established and can improve growth rates in several species, including swine [28–30]. The most important phenotypes for any antimicrobial feed additives are weight gain and feed efficiency. Studies using human lysozyme from transgenic goats’ milk did not show an improvement in growth of pigs consuming human lysozyme [8, 9]. This was likely due to the experimental design in these experiments as they were not conducted to evaluate the effect of lysozyme on pig performance. In these experiments, growth improvement due to lysozyme was likely masked due to the presence of antibiotics in both the control and the experimental diet [9]. Presumably, Maga et al. [8] fed diets that included antibiotics also. In addition, both Brudige et al. [9] and Maga et al. [8] fed dry, pelleted nursery diets in addition to the lysozyme-containing goats’ milk. Thus, it is unclear how much lysozyme was consumed by pigs in relation to the dry diets in these studies. Due to the changes in intestinal morphology and microflora, the pigs consumed a significant amount of lysozyme, but this amount may not have been sufficient to impact growth rate. Humphrey et al. [10] fed 152 mg human lysozyme (produced from transgenic rice) per kg feed, but did not improve the growth rate of chicks. However, the chicks had significantly improved feed efficiency over those reared on a diet containing neither the transgenic protein nor antibiotics.

**Fig. 1:**
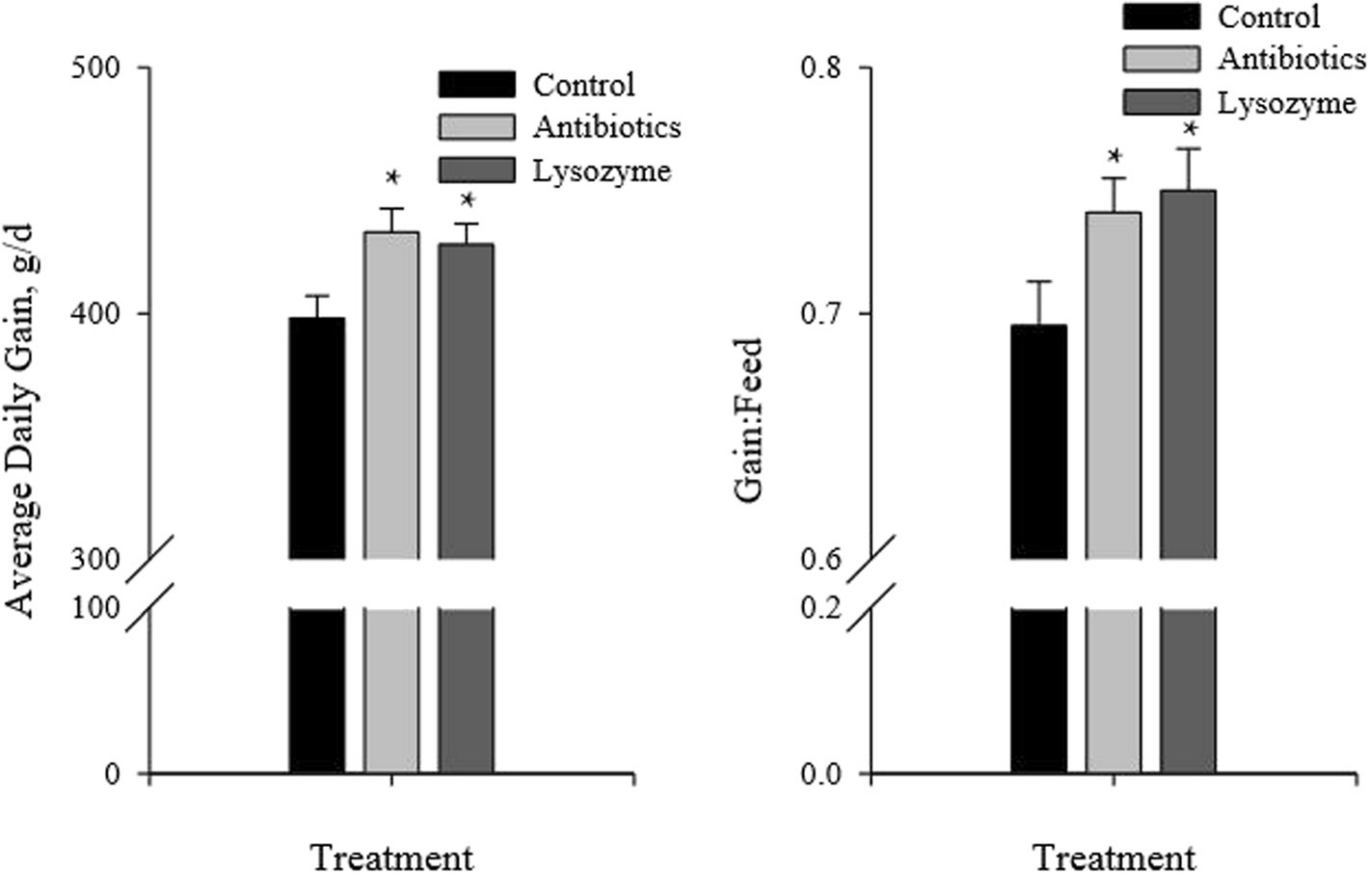
Average daily gain and feed efficiency of nursery pigs consuming control (non-mediated), control + antibiotics, or control + lysozyme diets for 28 days. Nursery pigs consuming lysozyme or antibiotics gained weight approximately 8 % faster. In addition, pigs consuming either lysozyme or antibiotics had improved feed efficiency of approximately 7 %. These data were adapted from Oliver and Wells [12] and Oliver et al. [13]. *Mean differs from control (*P* < 0.05)

Lysozyme sourced from chicken eggs improves growth performance comparable to neomycin/oxytetracycline (milk diets; [11]), carbadox/copper sulfate (nursery diets; [12]) or chlortetracycline/tiamulin hydrogen fumarate (nursery diets; [13]) compared with pigs consuming a nonmedicated diet (Fig. 1). Due to the study design, feeding group-housed pigs a milk diet, May et al. [11] did not have the statistical power to detect changes in feed efficiency. However, Oliver and Wells [12] and Oliver et al. [13] were the first examples of lysozyme improving feed efficiency in swine, where pigs consuming lysozyme had an improved feed efficiency of about 8 % compared with pigs consuming the untreated diet, which was similar to pigs consuming the antibiotic-treated feeds (Fig. 1).

### Gastrointestinal tract

Improved villus height and crypt depth in the small intestine generally indicates improved intestinal health [31–33]. However, due to the already rapidly changing gross morphology in nursery pigs due to weaning, observed changes in intestinal morphology due to dietary subtherapeutic antibiotic are variable. Studies have shown that some antibiotics improve morphology [12, 34] whereas others do not [30, 35]. Previous work with human lysozyme from transgenic goats’ milk or transgenic rice did not show improvements in intestinal morphology in the jejunum or ileum [9, 10, 36]. Cooper et al. [36] did show a tendency for lysozyme to increase duodenal villi height and observed a decrease in lamina propria thickness. Similar to the lack of improvement in growth performance in these studies, the lack of morphology response is likely due to the concomitant presence of antibiotics in the feed, or simply a lower consumption of lysozyme.

**Fig. 2:**
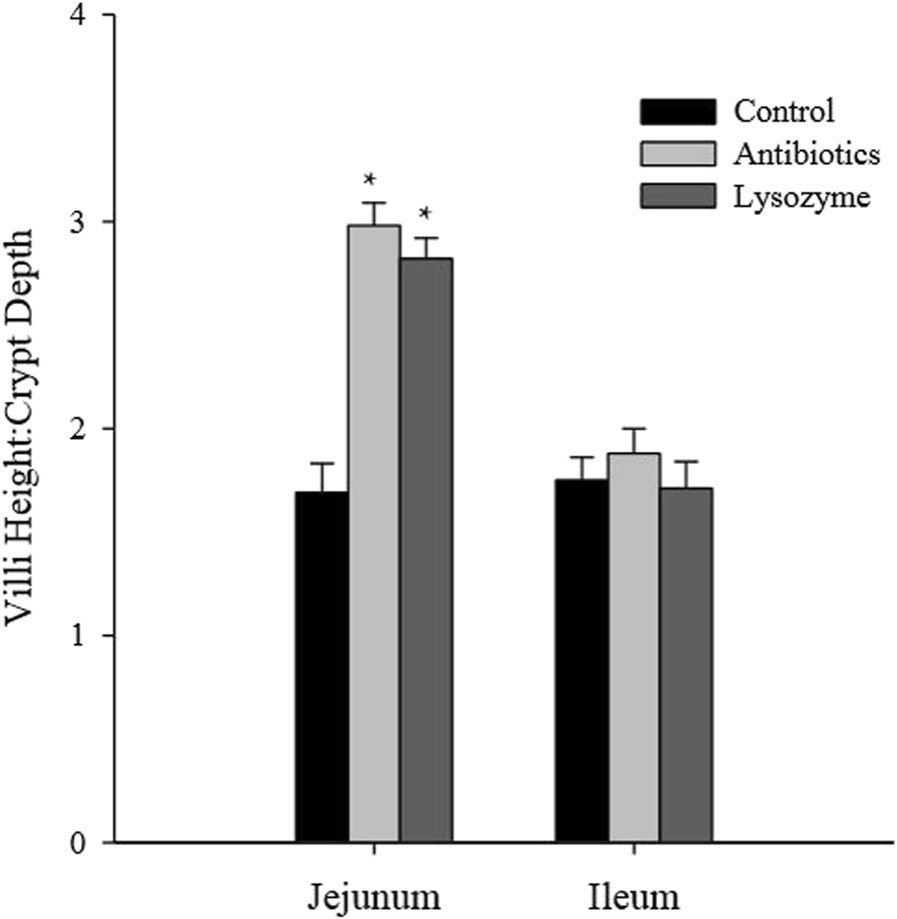
Villi height/crypt depth ratio of nursery pigs fed either a control (non-medicated), control + antibiotics, or control + lysozyme diet for 28 days. Villi height increased and crypt depth decreased exclusively in the jejunum of pigs consuming antibiotics or lysozyme, resulting in an increase of approximately 70 % in villi height to crypt depth ratio. These data were adapted from Oliver and Wells [12]. *Mean differs from control (*P* < 0.05)

May et al. [11] and Oliver and Wells (Fig. 2; [12]) both observed increased villus heights and crypt depths, indicating improved intestinal health. However, the major morphological responses in pigs consuming lysozyme or antibiotics in liquid diets was observed in the ileum [11] compared with responses seen exclusively in the jejunum by Oliver and Wells [12]. Presumably, this is due to the different physical forms of the diets consumed. Major changes occur in the gastrointestinal tract in response to the transition from a liquid to dry diet [37], in particular to ion transport [38]. Presumably the changes in structure and function of the small intestine allowed lysozyme and antibiotics to have a greater effect on the jejunum. Oliver and Wells et al. [12] observed decreased crypt depth in pigs consuming lysozyme or antibiotics (Fig. 2), whereas they were increased in pigs consuming lysozyme in liquid diets [11]. This is likely due to the fact that cellular proliferation is very high in the crypts in the younger animal, while villi enterocytes are longer-lived in suckling animals compared with weaned animals [39]. Nyachoti et al. [27] observed increased villi height in the ileum of pigs weaned at 17 days and fed an egg white source of lysozyme, but jejunum morphology was not measured. Changes in ileal morphology were likely due to the effect of the *Escherichia coli* K88 challenge on the small intestine [27]. Taken together, these data indicate that this source of lysozyme improves small intestinal morphology [11, 12, 27]. Improvements in small intestinal morphology may lead to a greater absorptive capacity and be a mechanism by which lysozyme and antibiotics improve growth rates.

### Metabolites

Nutritional regime, health status, age, level of production, and gastrointestinal microflora are a few examples of the many factors that contribute to the metabolite profile of an animal. It is clear that both lysozyme and antibiotics alter many of these factors including growth rate, microbiota (or at least individual organisms), and gastrointestinal health. Circulating urea N is a reliable indirect measurement to show the oxidation of dietary amino acids in young pigs [40, 41]. Blood urea N (BUN) is lower in pigs consuming either lysozyme or antibiotics under a chronic immune challenge compared with control pigs [13]. This contradicts earlier work in non-challenged pigs [12]. However, considering that pigs consuming lysozyme or antibiotics accrued more protein and consumed similar amounts of feed compared with control pigs [13], the greater BUN was expected. Therefore, presumably, pigs that consumed lysozyme or antibiotics utilized more of their dietary amino acids for protein deposition than control pigs. Oliver and Wells [12] likely had too few of animals to detect a response in BUN.

The most efficient way to measure metabolites is through metabolomic experiments. Brundige et al. [7] found 18 known serum metabolites that were changed by the consumption of lysozyme. Of these 18, most changed in a direction that was decidedly “positive” for pig health and(or) growth. Four of these (methionine, threonine, hydroxyproline, and urea) indicate a propensity for increased growth in pigs consuming lysozyme. Methionine, threonine, and hydroxyproline increased in serum indicating potential increases in protein synthesis and skeletal growth, while serum urea decreased. These findings support Oliver et al. [13], in that lysozyme consumption increased growth rate and decreased circulating urea, in addition to an increase in protein accretion compared with pigs consuming a non-medicated diet.

### Cytokines and Immune Response

Immune system activation, including pro-inflammatory cytokine and acute phase protein production, prevents animals from reaching their genetic growth potential [42]. For example, poultry and swine reared in germ-free environments grow at a faster rate than animals reared in conventional production environments [43, 44]. In addition, utilizing a clean vs. a dirty environment to stimulate a chronic immune response decreases animal performance [45–47]. In pigs, an immune response does not generally result in decreased feed conversion [48–50]. However, both lysozyme [12] and antibiotics [1] improve feed efficiency in nursery swine. In addition, Nyachoti et al. [27] reported that lysozyme alleviated the piglet response to an oral challenge of *Escherichia coli* K88, similar to traditional antibiotics.

While cytokines primarily regulate the immune response, they have an equal effect on nutrient metabolism. During an immune response, pro-inflammatory cytokines redirect nutrients away from growth and toward the immune response [51, 52]. Although not the only mode of action, cytokines increased both muscle protein degradation and acute phase protein production [53]. Cytokines and acute phase proteins were measured in a study designed to elicit a low level immune response, to both confirm the chronic immune stimulation and to determine the effect of antibiotics and lysozyme on the immune response [13]. Interleukin-6 and pig major acute phase protein were unaffected by immune status. In contrast, circulating levels of the cytokine tumor necrosis factor-α (TNF-α) and the acute phase proteins haptoglobin and C-reactive protein (CRP) were higher in chronically immune stimulated pigs compared with pigs reared in a clean nursery. These changes in cytokines and acute phase proteins, as well as the performance changes observed, indicate that an acceptable level of immune response was generated in pigs reared in the dirty nursery to make inferences into the effect of antibiotics and lysozyme on chronically immune stimulated pigs. Pigs consuming antibiotics or lysozyme had lower TNF-α, haptoglobin, and CRP, compared with control pigs, regardless of whether pigs were under chronic immune stimulation or reared in a clean nursery. Similarly, Lee et al. [54] observed lower haptoglobin levels in antibiotic-fed pigs compared with non-medicated controls. In addition, Nyachoti et al. [27] observed lower circulating TNF-α levels post-challenge in pigs consuming lysozyme. While these later studies used a different model (acute *Escherichia coli* challenges), antibiotics and lysozyme fed to pigs reduced the immune response when exposed to pathogens. In addition to these studies, Cooper et al. [36] determined that RNA for transforming growth factor-β1 was increased in unchallenged pigs consuming lysozyme from transgenic goats’ milk.

### Microbial ecology

It is clear that the microbiota are important to pig health and growth [26, 55]. However, Holman and Chenier [56] observed relatively minor changes to the pig’s microbiota in pigs consuming either tylosin or chlortetracycline. Unno et al. [57] showed that the use of antibiotics in swine feed inhibited potential pathogens. However, the use of chlortetracycline, sulfathiazole, and penicillin did not elicit a growth response making it impossible to determine if the change in microbiota was associated with improved performance. Clearly, more work in this area is warranted.

It is now well documented that lysozyme has antimicrobial qualities and improves pig performance and gastrointestinal health. It is likely that lysozyme alters the gastrointestinal bacterial population, either through direct bacterial elimination (Gram-positive bacteria) or changes to the ecology that favor one group of bacteria over another. However, little work has been done looking at the effect of lysozyme on pig gastrointestinal microbial populations. In a small, proof of concept experiment, Maga et al. [8] observed that lysozyme was capable of modulating the bacterial populations in the duodenum and ileum of both kid goats and piglets. In pigs, lysozyme from transgenic goats’ milk reduced both total coliforms and *E. coli* in the duodenum, while only total coliforms were reduced in the ileum. This small study clearly shows that lysozyme has the ability to alter microbial populations *in vivo*. Lysozyme was also shown to reduce enterotoxigenic *E. coli* (ETEC) in challenged piglets [27]. However, the observed effect of lysozyme on *E. coli* species seems to be variable. The prevalence of Shiga-toxigenic *E. coli* (STEC) is generally low in nursery pigs [20] and was not altered by lysozyme or antibiotics. The *eae* gene, which is an indicator gene for enteropathogenic and enterohemorrhagic *E. coli* (EPEC and EHEC, respectively) is observed in nursery pigs [20]. However, this gene increases over the course of the nursery phase, neither lysozyme or antibiotics seem to alter its abundance [20]. The different observations due to feeding lysozyme on *E. coli* may be due to the different sources of lysozyme, different species of *E. coli* (ETEC vs. STEC, EPEC, and EHEC), or the presence of a direct *E. coli* K88 challenge [27].

**Fig. 3:**
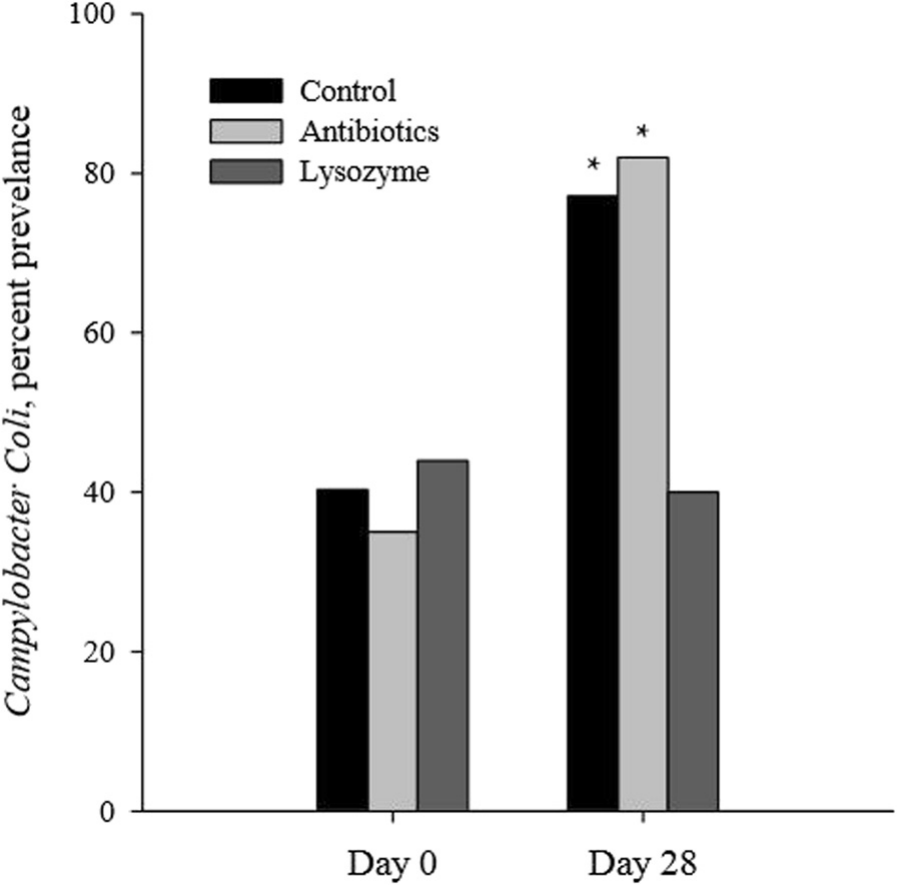
*Campylobacter* spp. shedding of nursery pigs fed either a control (non-medicated), control + antibiotics, or control + lysozyme diet for 28 days. Lysozyme, but not chlortetracyline/tiamulin in nursery swine feed prevented the normal increase in campylobacter shedding in the feces of nursery pigs. These data were adapted from Wells et al. [20]. *Within day, mean differs from lysozyme (*P* < 0.05)

Maga et al. [58] studied the microbiome of pigs consuming lysozyme expressed in transgenic goats’ milk. Lysozyme decreased the levels of *Firmicutes* and increased the levels of *Bacteroidetes* in pig feces. High levels of *Bacteroidetes* are associated with decreased nutrient absorption [59], but the level of change in piglets consuming lysozyme is unlikely to cause decreased absorption, especially considering the changes in gut morphology and performance observed when feeding lysozyme [12, 13]. At the taxonomic Family or Order level, lysozyme decreased the abundance of bacteria associated with disease (*Mycobacteriaceae*, *Streptococcaceae*, and *Campylobacterales*) and increased bacteria associated with gastrointestinal health (*Bifidobacteriaceae* and *Lactobacillaceae*). These data support May et al. [11] and Wells et al. (Fig. 3, [20]), who observed a 50 % reduction of *Campylobacter* spp. in pigs consuming lysozyme compared with non-medicated pigs. While carbadox/copper sulfate is effective against *Campylobacter* spp. [3], Wells et al. [20] observed that chlortetracycline/tiamulin hydrogen fumarate did not change the *Campylobacter* spp. in the feces similar to lysozyme.

## Conclusions

It is clear that feeding subtherapeutic levels of antibiotics improves performance and overall health and is used extensively throughout the swine industry. However, it is also clear that swine producers are under pressure to reduce or eliminate the use of antibiotics due to concerns over antibiotic resistance. Research into possible alternatives is essential and will allow swine producers to keep the animal well-being and monetary advantages of antibiotics without the perceived negative effects of their use. Lysozyme is a natural antimicrobial already used in other facets of the food industry. In nursery pigs, lysozyme added to feed improves gastrointestinal health, reduces potential pathogen shedding, and improves growth and feed efficiency. Therefore, lysozyme is a viable alternative to traditional subtherapeutic antibiotic use in swine production.

## Abbreviations

BUN: Blood urea nitrogen
CRP: C-reactive protein
TNF-α: Tumor necrosis factor- α
ETEC: Enterotoxigenic *E. coli*
STEC: Shiga-toxigenic *E. coli*
EPEC: Enteropathogenic *E. coli*
EHEC: Enterohemorrhagic *E. coli*
